# Does Disc Space Height of Fused Segment Affect Adjacent Disc Degeneration in Anterior Lumbar Interbody Fusion? A Radiological Study

**Published:** 2012-03-01

**Authors:** Sh Tang, W Xu

**Affiliations:** 1 Department of Traditional Chinese Medicine, Medical School, Jinan University, Guangzhou, China; 2 Department of Orthopaedics Surgery, Tianjin Hospital, Tianjin City, China

**Keywords:** Adjacent segment degeneration, Anterior lumbar interbody fusion, Disc space height, Segmental lordosis, Whole lumbar lordosis

## Abstract

**Background:**

The restoration of disc space height (DSH) is essential in anterior lumbar interbody fusion (ALIF), while it is unclear whether the reduction of DSH may alter the mechanical status and adversely affect adjacent segment, and few literatures focused on the subject.

**Methods:**

Ninety five patients who had undergone ALIF for degenerative disc disease at our institution between March 2004 and March 2007 were retrospectively reviewed and 76 patients were enrolled in this study. Preoperative, postoperative and the final follow-up segmental lordosis (SL), whole lumbar lordosis (WLL) and DSH were measured and compared in adjacent segmental degeneration (ASD) group and non-ASD group, and the relationship between DSH, SL, WLL and ASD were investigated retrospectively.

**Results:**

In 76 patients, the radiographic ASD was proven in 25 (32.9%) and symptomatic ASD in 2 patients. There was a significant correlation between DSH and SL, but was insignificant between DSH and WLL, and a significant correlation was noticed between ASD and SL, WLL and DSH at final follow-up.

**Conclusion:**

The normal DSH and SL is important for preventing ASD and an anterior cage with appropriate height and lordotic angle to be used in ALIF to maintain the proper DSH and SL.

## Introduction

Anterior lumbar interbody fusion (ALIF) has been widely used to treat a variety of spinal diseases, by distracting the affected degenerated segment, placing the interbody fusion devices and stabilizing the motion segment. The restoration of disc space height (DSH) is essential in ALIF and the decrease of DSH of fused segments, one of the common complications, may alter the mechanical status and adversely affect the clinical result.[1][2][3][4] Moreover, we speculate the decrease of DSH may change the sagittal alignment, change the stress conduction and aggravate the adjacent segment degeneration (ASD).

Many authors focused on the correlation between ASD and abnormal sagittal lumbar alignment. Umehara et al.[5] studied the effect of sagittal malalignment after transpedicular posterolateral fusion in human cadavers and suggested altered biomechanics involving increased loading of the posterior column and posterior shear force at the proximal adjacent segment. Kumar et al.[6] further studied the patients who had undergone instrumented posterolateral fusions and found very high rates of ASD when alterations of abnormal sagittal alignment were present. Akamaru et al.[7] reported that hypolordotic alignment of the fused segments caused the greatest amount of flexion-extension motion at the superior adjacent segment, and suggested that this was related to ASD, emphasizing the importance of maintaining normal segmental lor-dosis (SL) in lumbar interbody fusion. However, up to now, few literatures were available focused on the correlation between DSH and ASD in ALIF.

Therefore, we reviewed retrospectively 95 patients who underwent ALIF at our institution between March 2004 and March 2007. The purpose of this study was to (1) determine the correlation between DSH and SL as well as whole lumbar lordosis (WLL), and (2) determine if the decrease of DSH may aggravate ASD in ALIF.

## Materials and Methods

Ninety-five patients who had undergone ALIF for degenerative lumbar diseases at our institution between March 2004 and March 2007 were reviewed retrospectively. We selected the procedures performed at single-level and augmented with pedicle screws. To facilitate the study, we excluded patients with previous spine surgeries, concomitant scoliosis of more than 15 degrees, a compression fracture or instability at the adjacent segment, those who had undergone simultaneous decompression at adjacent segments, and those who did not obtain solid fusion after surgery. Of 95 patients, 76 patients were enrolled in the study. There were 45 males and 31 females with an average age of 45.2 years (range from 35.3 to 58.5 years). The mean follow-up period was 47.8 months (ranging from 38 to 76 months). The preoperative diagnoses were as follows: degenerative spondylolisthesis (grade I or II) in 7 patients, discogenic pain in 33, and segmental instability in 36 patients. In all cases, anterior stand-alone cage was used and pedicle screw fixation was performed to augment ALIF. The fused segments were L4-5 in 49 and L5-S1 in 27 patients. The informed consent was obtained from each patient included in the study and the study protocol conforms to the ethical guidelines of the 1975 Declaration of Helsinki as reflected in a prior approval by the institution's human research committee.

**Fig. 1:**
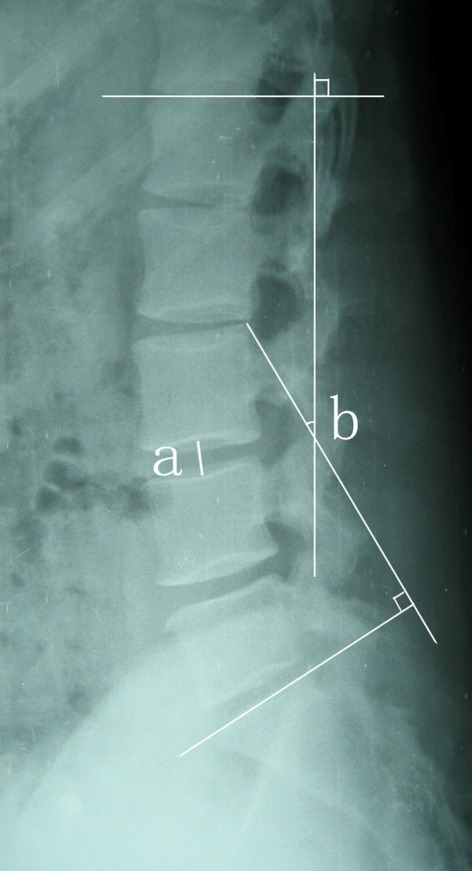
The DSH (a) was determined to be the distance from the midpoint of the anteroposterior diameter of the inferior endplate to the superior endplate. The whole lumbar lordosis (WLL), (b) was defined as the angle subtended by the superior endplate line of L1 and the superior endplate line of S1.

**Fig. 2:**
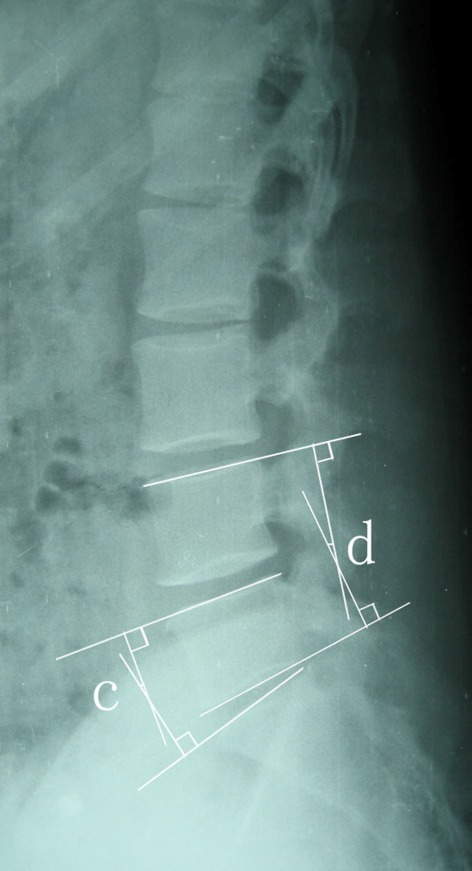
The segmental lordosis (SL) at L4-5 (d) was defined as the angle subtended by the superior endplate line of L4 and the inferior endplate line of L5. The SL at L5-S1 (c) was defined as the angle subtended by the superior endplate line of L5 and the superior endplate line of S1.

The radiographs of all patients were evaluated preoperatively, postoperatively and at the final follow-up with particular focus on the fusion status, WLL, SL and DSH at fused segment. An independent observer made radiographic assessments. The outcome was evaluated from anteroposterior, lateral, and flexion-extension radiographs and DSH, SL, WLL were used as parameters reflecting sagittal alignment on plain radiographs. The measurements were performed twice for each parameter with an adequate time interval in order to prevent bias from distorting the results and the average values of the two measurements were used for the evaluation. Interbody fusion was determined to be achieved if a transvertebral osseous bridge had formed anterior and posterior to the cage on the plain radiographs, if a radiolucent line between the cage and endplate was not present, if loosening or breakage of pedicle screws did not occur and if there was no motion on dynamic flexion-extension radiographs.[8] In the measurement, the WLL was defined as the angle subtended by the superior end plate line of L1 and the superior end plate line of S1.[9] The DSH was determined to be the distance from the midpoint of the anteroposterior diameter of the inferior endplate to the superior endplate8 (Figure 1). The SL at L4–L5 was defined as the angle subtended by the superior end plate line of L4 and the inferior end plate line of L5. The SL at L5–S1 was defined as the angle subtended by the superior end plate line of L5 and the superior end plate line of S19(Figure 2). The measurements were made using image J software (version alpha 4.0.3.2; http:// www. scioncorp.com). With the aid of computer guidance, all images were corrected for radiographic magnification error. Standard implant reference points and implant dimensions were used to determine the percentage of magnification error.[10]

The degree of disc degeneration at adjacent segments was rated from grades 0 to 3 using the classification system of Wilke[11] (Table 1). ASD was diagnosed when plain radiographs demonstrated one or more of the following lesions at the segment adjacent to the fused segment that were not present preoperatively: more than 4 mm of anterolisthesis or retrolisthesis, more than 10 degrees of angular motion between adjacent vertebral bodies on flexion and extension radiographs, spinal stenosis caused by facet joint hypertrophy, degenerative scoliosis, more than 10% loss of disc height, and more than 3 mm of osteophyte formation.[9][12][13] Radiographic evidence of ASD without clinical symptoms was defined as radiographic ASD, whereas aggravated ASD with clinical symptoms was defined as symptomatic ASD.

**Table 1:** Radiographic grading system for lumbar intervertebral disc degeneration

**Height loss**	**Osteophyte formation**	**Diffuse sclerosis**	**Overall degree of degeneration**
Anterior and posterior height loss with respect to the individual height before degeneration	Sum of points of eight edges	Sum of points of both adjacent vertebral bodies	Sum of points of "Height Loss", "Osteophyte Formation" and "Diffuse Sclerosis"
	No osteophytes: 0 points	No sclerosis: 0 points	
	<3 mm: 1 point	0.25 partially or completely affected: 1 point	
	≥3 mm but <6 mm: 2 points	0.5 partially or completely affected: 2 points	
	≥6 mm: 3 points	>0.5 partially or completely affected: 3 points	
0=0%	0=0 points	0=0 points	0 point = grade 0 (no degeneration)
1=<33%	1=1–8 points	1=1–2 points	1–3 points = grade 1 (mild degeneration)
2=≥33 but <66%	2=9–16 points	2=3–4 points	4–6 points = grade 2 (moderate degeneration)
3=≥66%	3=17–24 points	3=5–6 points	7–9 points = grade 3 (severe degeneration)

The patients were divided into two groups during follow-up according to the development of ASD. ASD group included patients who were noted at last follow-up to have ASD. Non-ASD group included patients who were not noted to have ASD. To investigate the lordosis change, preoperative, postoperative and last follow-up standing lateral x-ray views were evaluated using the Cobb angle method. Through measurement of their radiographs, the preoperative, postoperative and last follow-up SL, DSH and WLL were obtained and compared in the two groups. In addition, the relationship between DSH, SL, WLL and ASD were investigated retrospectively.

Statistical analysis was performed using SPSS software (Version 17.0, Chicago, IL, USA). The difference of variables before operation, after operation and at final follow-up were analyzed using paired sample t-test, and the difference between groups was compared using independent 2-sample t test or Chi-Square test, and Pearson’s correlation analysis was used to investigate the correlation of DSH, SL and WLL, and binary logistic regression analysis was used to determine the risk factors related to ASD. A p value less than 0.05 was considered to indicate statistical significance.

## Results

In 76 patients, the radiographic ASD was proven in 25 (32.9%) of 76 patients and the diagnosis of ASD were as follows: anterolisthesis in 4 patients, retrolisthesis in 3 patients, angular instability in 5 patients, spinal stenosis in 4 patients, disc height loss (>10%) in 6 patients and osteophyte formation (>3 mm) in 3 patients. In terms of the fusion level, ASD at L4-5 was observed in 19 of 49 patients (38.8%) and at L5-S1 was noticed in 6 of 27 patients (22.2%) (p=0.14). Two patients (2.6%) were diagnosed as symptomatic ASD. There was no significant difference in sex (p=0.69), age (p=0.46) and the durations of the radiographic follow-up periods (p=0.37) between the ASD and non-ASD groups.

Table 2 presents the preoperative, postoperative and final follow-up data for patients in ASD and non ASD groups. There were no significant intergroup difference in the mean of SL, DSH and WLL preoperatively (p=0.87, 0.24 and 0.73, respectively) and postoperatively (p=0.41, 0.36 and 0.93, respectively). However, the mean of decrease of WLL was 2.7° in ASD group and 0.9° in Non-ASD group and the value was greater in ASD group (p=0.04). At the same time, the mean of the decrease of SL was 3.5° in ASD group and 0.7° in non-ASD group, and there was significant difference between the two groups (p=0.001). In terms of DSH, the mean of decrease of DSH was 3.9 mm in ASD group and 1.2 mm in Non-ASD group, and there was a significant difference between two groups (p=0.001).

**Table 2:** Comparison between ASD and Non-ASD groups

	**ASD (n=25)**	**Non-ASD (n=51) **	***P *value**
Disc Space Height^a^ (DSH, mm)			
Preoperative	9.0±3.9	9.2±3.5	0.24
Postoperative	14.1±2.7	14.6±4.8	0.36
Final follow up	10.7±4.2	13.7±3.6	0.00
Decrease of Disc Space Height	3.9±2.1	1.2±1.6	0.00
Segmental Lordosis (SL, Deg)			
Preoperative	17.1±7.1	17.4±6.5	0.87
Postoperative	19.5±5.7	20.1±6.3	0.41
Final follow up	16.7±3.9	19.4±6.1	0.01
Decrease of Segmental Lordosis^b^	3.5±1.4	0.7±0.3	0.00
Whole Lumbar Lordosis^c^ (WLL, Deg)			
Preoperative	47.8±13.2	51.6±14.5	0.73
Postoperative	54.3±13.9	58.1±16.4	0.93
Final follow up	51.6±14.7	57.3±15.2	0.00
Decrease of Whole Lumbar Lordosis	2.7±0.8	0.9±1.3	0.04

^a^ Decrease of Disc Space Height=Postoperative DSH-final follow-up DSH

^b^ Decrease of Segmental Lordosis= Postoperative SL-final follow-up SL

^c^ Decrease of Whole Lumbar Lordosis=Postoperative WLL-final follow-up WLL

Compared to the preoperative values, the mean of SL and WLL at final follow-up indicate a significant increase in both ASD group (p=0.02, p=0.04, respectively) and non-ASD group (p=0.01, p=0.03, respectively). In terms of DSH, the values were significantly higher in non-ASD group at final follow-up than those in preoperative examination (p=0.00). However, compared to the preoperative examination, the values in ASD group was insignificant (p=0.054) at final follow-up. In addition, there was a significant correlation between DSH and SL (Pearson’s coefficient=0.716; p=0.03), but insignificant between DSH and WLL (Pearson’s coefficient=0.265; p=0.16)

The contributions of potential risk factors for the development of ASD were summarized in Table 3. The DSH, SL and WLL did not have statistically significant values in both preoperative (p=0.82, 0.77 and 0.59, respectively) and postoperative examination (p=0.37, 0.46 and 0.33, respectively), while at the final follow-up, the three parameters (p=0.04, 0.03 and 0.02, respectively.) were risk factors for ASD (Table 3).

**Table 3:** Association of ASD with risk factors by binary logistic regression

	***P* value**	**Odds Ratio (95%CI)**
Segmental lordosis (SL)		
Preoperative	0.87	0.98 (0.79-1.23)
Postoperative	0.56	1.07 (0.81–1.35) (0.83–1.34)
Final follow-up	0.03	71.38 (2.49-3124.43)
Whole lumbar lordosis (WLL)		
Preoperative	0.69	1.02 (0.93–1.15) (0.92–1.12)
Postoperative	0.43	0.96 (0.86–1.07) (0.86–1.07) (0.86–1.07)
Final follow-up	0.02	55.36 (1.32–2869.51) (1.11–2966.52) (1.11–2966.52)
Disc space height (DSH)		
Preoperative	0.92	1.79 (0.06–64.82)
Postoperative	0.77	1.18 (0.01-1.96)
Final follow-up	0.04	68.76 (3.25-2543.97)

## Discussion

In the present study, we reviewed 76 patients treated at our institution and embarked a study on the correlation between DSH and ASD in ALIF. To the best of our knowledge, few studies have been published on the subject in English literatures. In terms of ASD, although some scholars claimed that it followed a natural degenerative course in patients who were predisposed; most scholars insisted that altered biomechanics appeared to play a key role in its development. At the same time, some suggested that ALIF may reduce damage to the integrity of the posterior complex, restore normal lordosis to correct the spinal malalignment,[14] offer a protective effect on the adjacent level stress[13][15] and lower the rate of ASD.[16] In the present study, ASD was observed radiographically in 25 (32.9%) out of 76 patients. While, the reported rates using radiographic criteria vary from 8% to 100%.[13] The ASD rate in the present study was relatively low, indicating the advantage of ALIF in protecting adjacent segment from ASD. In addition, the patients in the present study lied within a young and narrow spectrum of age distribution (36.7-51.8 years), which made it difficult to adduce age as a risk factor for ASD.[9]

We found SL and WLL at final follow-up to have closely correlation with ASD, which confirmed the viewpoint of many authors.[9][13][15] In addition, 23 (92.0%) out of 25 patients in ASD group had decreased DSH, and 18 (35.3%) of 51 patients in non-ASD group at last follow-up had decreased DSH and the difference between two groups was significant. Furthermore, the decrease of DSH was 1.2±1.6 mm in non-ASD group, and 3.9±2.1 mm in ASD group and the value in ASD group was significantly higher than that in non-ASD group, indicating that DSH may be closely correlated with ASD.

Moreover, some patients in two groups sufferred from degenerative disc disease and their intervertebral discs were degenerative and DSH decreased preoperatively. While, some were discogenic pain patients and there were no apparent degenerative changes in intervertebral discs, and their DSH was nearly normal, which resulted in the relatively high values of DSH in two groups preoperatively, and there was no significant correlation between preoperative DSH and ASD, and even, there was no significant difference between the final follow-up and preoperative values in ASD group. However, in the non-ASD group, there was significant difference between the preoperative and final follow-up DSH. In addition, the results of the current study demonstrated that there was significant correlation between DSH at final follow-up and ASD. Subsequently, we suggested DSH at final follow-up to be a risk factor for the developing of ASD.

Some authors evaluated the correlation between SL and DSH. Using biomechanical study on ProDisc-L implants, Gaffey[17] concluded that there was significant correlation between implant height and SL and an increasing implant height produced a significant increase in SL. In the report of twenty-six patients undergone transforaminal lumbar interbody fusion (TLIF), Kim[8] confirmed a loss of SL to have a significant correlation with the decrease of DSH resulting from cage subsidence. In ALIF cases, the posterior elements and facet joints were usually intact, normal and stable, together with the fixation of posterior instrumentation, the height of posterior structure may keep unchanged and only DSH may be decreased while cage subsidence occur. Subsequently, we believe that the decrease of DSH lead to a decrease in SL. At the same time, our results proved aforementioned assumption. Nevertheless, we did not find any significant correlation between DSH and WLL, which may be attributed to the small sample size in the present study. In addition, the standing posture of patients may have more influence on WLL, which may also affect the results. As many authors suggest that the abnormal SL may adversely affect the stress conduction at the adjacent segments, the effect of DSH on the ASD may be attributed to the changed sagittal alignment indirectly.

In the study of Bae,[9] 103 patients underwent ALIF or TLIF for the treatment of adult low-grade isthmic spondylolisthesis that were retrospectively reviewed with a minimum of a 36-month follow-up period, and the postoperative SL, preoperative SL, WLL were regarded as significant risk factors for ASD, which are inconsistent with our conclusion. In the present study, ALIF was performed in all 76 patients. While in Bae's study, instrumented ALIF and TLIF were performed, respectively, in 75 patients and 28 patients. TLIF, differing from ALIF in surgical techniques, may have different influence on adjacent segments. Moreover, the follow-up period in Bae's study was different from the present study, which may also affect the final conclusion.

At the same time, in present study patients with L5-S1 fusion showed a relatively lower rate for developing of ASD than patients with L4-5 fusion. Although the difference was not significant, the results may be in accordance to the report by Quinnell[18] and Alexander.[19] They attributed the higher rate of ASD in L4-5 fusion to the mechanical reasons based on the enhanced loads that act on this segment. The intervertebral shear force and the highest range of motion for flexion/extension movements in L4-5 segment was larger than other lumbar segments, and when the segment fused, the adjacent levels have to compensate the generated segmental immobility and result in more severe ASD.

There are some limitations in the present study. We performed a study between DSH and radiographic ASD, while the correlation between symptomatic ASD and DSH is unknown. Moreover, the sample size is relatively small and the period of follow-up is short, and a prospective trial studied during a long term follow-up would certainly provide more useful information. While, we can conclude that there is close correlation between DSH and SL, and decreased DSH may adversely affect the adjacent superior segment and aggravate ASD in ALIF. Therefore, it is believed that care and attention should be given to introduce DSH in normal range, and for this purpose, it is recommended that an anterior cage with appropriate height and lordotic angle be used in ALIF to maintain the proper DSH and SL.
